# A Path Toward Inclusive Social Cohesion: The Role of European and National Identity on Contesting vs. Accepting European Migration Policies in Portugal

**DOI:** 10.3389/fpsyg.2020.01875

**Published:** 2020-08-14

**Authors:** Isabel R. Pinto, Catarina L. Carvalho, Carina Dias, Paula Lopes, Sara Alves, Cátia de Carvalho, José M. Marques

**Affiliations:** Center for Psychology at University of Porto, University of Porto, Porto, Portugal

**Keywords:** national and European identity, pro-inclusion vs. contestation, immigration policies, social inclusion, political tendencies

## Abstract

The Western hemisphere has witnessed recent increased immigration flows generating social and political debate across Europe. In one view, migration flows represent an opportunity to construct a diverse social cohesion. In another view, migration flows are perceived as a threat to existent national cultures. This view is held by political nationalisms and right-wing populist forces installed in the majority of EU countries’ parliaments, accentuating discrimination against immigrants and residents in Europe. We theorize that European identity predicts positive attitudes toward immigrants (prosocial behavior and support for inclusive policies), whereas national identity’s predictions of attitudes toward immigrants’ inclusion depends on participants’ political tendency. Moreover, we test the mediation effect of positive (humanitarian concerns and economic benefit) and negative (jobs scarcity, cultural deterioration, and invasion) arguments used in political discourses regarding immigrants’ inclusion on the relation between national and European identities and attitudes toward immigrants’ inclusion. Results (Portuguese sample, *N* = 176) show that national identity predicts negative attitudes toward immigrants’ inclusion, but only among right-wing individuals. Among left-wing individuals, national identity predicts less contestation to immigrant’s inclusion sustained by humanitarian concerns. Interestingly, European identification weakened right-wing individuals’ adherence to discriminatory arguments and increased perceived economic contribution that immigrants bring to society, increasing agreement with prosocial behavior and immigrants’ inclusion. We discuss that European identity, sustained in humanitarian values and economic benefit, may stimulate a stronger multicultural social cohesion, intergroup trust, and social well-being based on democratic values, social justice, and equality, and on the respect for human dignity.

## Introduction

Europe has been one of the leading destinations for immigration, with an increased migratory flow over the past decade (e.g., de Wenden, 2019). The motivations and reasons for migration are diverse, such as education purposes, work, family reunification, or fleeing from war or conflict in their native countries (European Commission, 2014). For the host countries, this increased migration flow has represented both an opportunity (e.g., compensation for the worker shortage in some professional areas, counterbalance of the demographic aging, cultural diversity) and a challenge (e.g., need for immigrants’ integration programs, regulatory migration laws and procedures, strategies to counteract discrimination, and intolerance toward immigrants).

The 2008 global economic and financial crisis and the consequent increase in unemployment, poverty, and social instability led many citizens to leave their nations (those most affected by the crisis) and seek better opportunities in other countries (Thyssen, 2018; de Wenden, 2019). When Europe was still recovering from the economic crisis, the refugee crisis escalated in 2015, increasing the number of migrants arriving in the European Union (EU) from the Mediterranean Sea and through Southeast Europe. To deal with the biggest migration flow since World War II, the European Commission has implemented the European Agenda on Migration (European Commission, 2015). This set of political measures, based on solidarity and shared responsibility (European Commission, 2019b), is grounded on four pillars: reduction of incentives for irregular migration; strengthening of borders, protection and asylum; and integration and legal migration (e.g., Apap et al., 2019; European Commission, 2019a). Consistent with the inclusive European Agenda, many political leaders across Europe publicly advocated for inclusiveness and integration, calling for open borders and humanitarian actions to save lives. One of these leaders was Chancellor Angela Merkel, who opened Germany to host up to 1 million refugees (La Baume, 2017). However, pro-inclusion orientations and policies were not accepted by all countries and political parties, and thus do not necessary lead to an effective integration attitude and societal well-being. Indeed, a general *social malaise* (perceived anomie, lack of trust in political elites, feelings of social decline, and ethnocentrism) seems to be rising in contemporary Europe with immigrants’ entrance (Aschauer, 2014).

The 2008 crisis and the intense immigration inflow increased cultural and economic security concerns, fueling the debate on free movement, national security, border control, and EU and national migration policies (Wodak and Boukala, 2015). Some political leaders potentiated public contestation against migrants, leading to the surge of new support to right-wing parties across Europe (Volkan and Fouler, 2009; Davis and Deole, 2017). The intensification of the migratory flow was proceeded by the rise of Euroscepticism, populist rhetoric, and right-wing leaders with strong anti-immigration positions across Europe (Torreblanca and Leonard, 2013; Dennison and Dražanová, 2018; Ruedin and van Heerden, 2018). Their arguments have focused, essentially, on the need to protect their countries against “invasion” and “loss of identity,” and to defend the interest of nationals against the perceived negative consequences caused by immigrants, such as job scarcity, burden on the social and healthcare systems, unfair redistribution of financial resources, increased criminality and terrorism, and cultural deterioration (e.g., Volkan and Fouler, 2009; Ruedin and van Heerden, 2018; de Wenden, 2019). For example, Viktor Órban, campaigning for the third term as Hungary prime minister in 2018, claimed that Europe was under invasion and that Brussels (i.e., the European Union) was not defending their people (Walker, 2018). In 2018, the President-elect in Czechia, Miloš Zeman, also argued that Europe was under invasion by Muslim immigrants and adopted a Eurosceptic campaign and an anti-immigration position, criticizing EU refugee policies (Dražanová, 2018). In France, Marine Le Pen, during the Presidential election campaign, also claimed that she would protect France through closing borders policies to protect against jobs scarcity and the terror threat allegedly posed by migrants (Dearden, 2017). She also argued that immigrants are draining resources, rejecting French values, and deteriorating their culture, emphasizing that French identity is in decline (Cooper, 2017; Atabong, 2018). Former Italian Minister of the Interior Matteo Salvini spoke about “cultural war,” arguing that Islam and Muslims’ values are not compatible with Europeans’, and claiming that immigrants pose a symbolic (cultural) threat (King, 2017). Other threatening rhetoric comes from the United Kingdom. For instance, the current leader of the Brexit Party, Nigel Farage, has been claiming that immigrants would take British people’s jobs and undermine their cultural values (BBC, 2015). In Germany, the populist rhetoric, anti-EU, and anti-immigrant positions also have been increasing. For example, Tony Gentsch, from the radical right-wing *Der Dritte Weg* party (“The Third Way”), claims that the country should put Germans’ interests first, close the borders, and stop the “foreign infiltration” (MacGregor, 2019). In the Netherlands, Geert Wilders, founder of the nationalist, right-wing, populist Party for Freedom, claimed that Islamization is the biggest problem of the country, representing a threat to their identity and freedom (Damhuis, 2019).

In Portugal, although there is an evident forthcoming of this growing trend of nationalism and anti-immigration sentiments across Europe, the situation is quite different so far (Wise, 2019). Indeed, Portugal is officially in line with EU recommendations on migration and maintains a tolerant, open, and positive attitude toward immigrants (Wise, 2019). In fact, the Portuguese Government promotes inclusive immigration policies and publicly debunks anti-immigration myths with facts, which is a fundamental promoter of citizens’ pro-immigrant attitudes (Visintin et al., 2018). For example, the Minister of the Presidency, Mariana Vieira da Silva, has emphasized on several occasions that instead of being a burden to Portuguese economy, immigrants contribute positively to taxation because their contributions are greater than the social benefits they receive, they represent demographic advantages (in counterbalancing demographic aging), and fill jobs with a shortage of workers; thus, they have a benefic impact to the country’s development (e.g., República Portuguesa, 2019; MadreMedia, 2019). Also, according to the current Portuguese Prime Minister, António Costa, Europe needs to mobilize against populism and xenophobia, emphasizing that immigration is essential to counter the demographic aging (Wise, 2019). Consistently, the current President of the Portuguese Republic, Marcelo Rebelo de Sousa, stated that Portugal intends to maintain an open door policy (ONU, 2019) and will continue to defend their inclusive policies and improve migrants’ full integration (Coelho, 2019).

Despite the pro-immigrant orientation of Portuguese political leaders, one right-wing deputy (leader of the CHEGA Political Party) was elected in the 2019 Portuguese Parliamentary elections (in fact, consistently with the anti-immigration trend across Europe). So far, his positions have been controversial and condemned by all the other political leaders. For example, recently he publicly stated that a black female Portuguese deputy should return to her “home country” (Penela and Lusa, 2020). Immediately, he was condemned by the President of the Portuguese Assembly of the Republic, with the full support of all other political parties with parliamentary representation (Gonçalves, 2020). Nevertheless, support for this party has been rising since the 2019 elections (it obtained around 1.2% of votes) and is currently achieving near 6% of people voting intentions (Dinis and Rosa, 2020).

### Social Polarization and Immigration Contestation

Notwithstanding the EU efforts and its pro-inclusion orientation and policies, there has been a lack of consensus among the European countries on what should be the best strategy to respond to the migrants’ flow. Indeed, immigrant inflows have led to opposing views and positions on migration policies and border control among the member states. Thus, as a result of this lack of consensus, the EU project on migration is facing contestation from European leaders, governments, citizens, and organizations across Europe. As a result, we have been observing, across many European countries, the occurrence of anti-immigration protest behavior (e.g., protests in Chemnitz, German, August 2018; and in Brussels, Belgium, December 2018). Nevertheless, because anti-immigration politicization is growing and pro-immigration political statement is less and less granted, pro-immigration popular demonstrations are also being organized and taking place to oppose and counteract the European right-wing nationalism trend (e.g., march against Italy’s new anti-migration law, December 2018; the Sardines movement against populism, racism, and the anti-immigration wave, in Bologna, Italy, in November 2019).

Following the growing divergences and conflicts resulting from the ever-rising inflow of migrants, occurring both within and between EU countries, studies in the social psychology domain have aimed to understand what is fueling the negative and the positive attitudes of the EU’s citizens toward migrants, leading them to support vs. reject pro-immigration policies and get involved in contestation vs. prosocial behaviors.

### Antecedents of Anti- and Pro-immigration Attitudes

#### Perceived Threat vs. Positive Contributions Associated With Immigrants

Perceived threat is one of the most common correlates of negative attitudes toward immigrants, specifically realistic (threats to the ingroup material and economic resources) and symbolic threats (threats to the ingroup values, beliefs and norms; e.g., Stephan et al., 2005; Sides and Citrin, 2007; Green, 2009; Murray and Marx, 2013). Perceived threat has also been shown to mediate the relationship between prejudice and support for discriminatory behavior against immigrants (Pereira et al., 2010), indicating that perceived threat might be a relevant argument to reinforce the link between prejudice and actual discrimination, thus contributing to the rise of ethnocentrism, mistrust in immigrants, and decreasing social well-being (Aschauer, 2014).

Many studies focus on the impact of perceived immigration economic costs vs. benefits on discrimination. As mentioned above, it has been found that nationals are fearful that immigrants may increase labor market competition and be a burden to public finances by depending on the welfare state, which obviously leads to less favorable attitudes toward immigrants (e.g., Dustmann and Preston, 2004; Harell et al., 2012; Hatton, 2016; Meuleman, 2018; Edo et al., 2019). On the other hand, those who perceive immigrants to contribute to the host country’s economy express more positive views about of immigration (Esses et al., 2001; Haubert and Fussell, 2006; Rouse et al., 2010; Reitz, 2012; Ekins, 2013).

#### National Identity and Nationalisms

Evidence from opinion polls showed that stronger nationalist sentiments are associated with greater opposition to immigration (Dennison and Dražanová, 2018). This is not surprising given that nationalism reflects individuals’ beliefs in the superiority of their nation in situations of perceived competition over scarce resources (e.g., job, economic benefits) associated with outgroup derogation, rejection, and hostility (e.g., Baughn and Yaprak, 1996; Mummendey et al., 2001; Osborne et al., 2017).

In the same vein, research has shown that the stronger individuals’ national identification, the more they hold negative attitudes toward immigrants (e.g., Blank and Schmidt, 2003; Esses et al., 2004, 2006; Yitmen and Verkuyten, 2018). This association is explained by threats that migrants are perceived to pose to the national population (e.g., Yitmen and Verkuyten, 2018). Thus, commitment to the nation in this sense involves a protective strategy that implies discrimination against migrants.

#### A Superordinate Identity

According to social identity and self-categorization theory (e.g., Tajfel and Turner, 1986; Turner et al., 1987), individuals belong to multiple social groups and each can be more or less salient depending on the context. In addition, people can categorize at different levels of abstraction, being able to define themselves as members of (sub)groups (e.g., nationality) and as members of more inclusive high-order system-level (or superordinate) groups (e.g., European or global identity) (Reynolds et al., 2013; Reysen and Katzarska-Miller, 2017). Thus, individuals can recategorize themselves and others (e.g., foreign citizens) to be part of a broader ingroup, which in turn leads to more positive attitudes toward them (see common ingroup identity theory; Gaertner and Dovidio, 2000; Sparkman and Eidelman, 2018). Indeed, greater identification with a more inclusive superordinate category (e.g., global citizen) or strong supranational attitudes increases prosocial value and behaviors (Reysen and Katzarska-Miller, 2013; see also Peitz et al., 2018).

#### Humanitarian Values

Those citizens that are more focused on humanitarian concerns are more likely to hold positive behavioral intentions toward immigrants: “Humanitarian concerns involve a sense of compassive care and moral responsibility for the welfare of fellow human beings, especially when they are in need (…) and are based on a shared humanity” (Yitmen and Verkuyten, 2018, p. 233). These concerns may lead to the defense of pro-inclusion policies, more solidarity toward immigrants, and more positive behavioral intentions (e.g., volunteering, pro-immigrant activism, money donation) (e.g., Karakayali, 2017; Kleres, 2018; Milan, 2018), thus promoting stronger pro inclusive behavior and societal well-being.

Moreover, the endorsement of humanitarian values can attenuate feelings of threat felt by the national majority, as these values relate to “a sense of concern for the welfare of fellow human beings, and leads to the belief of personal responsibility to help those who are in need” (Newman et al., 2015, p.4). Indeed, there is evidence that humanitarian values are negatively associated with anti-immigration attitudes (Cowan et al., 1997; Oyamot et al., 2006, 2012; Newman et al., 2015; Verkuyten et al., 2018) unless people feel that immigration threatens such values (Kende et al., 2019). The endorsement of values, such as universalism, defined as being the “understanding, appreciation, tolerance, and protection for the welfare of all people and for nature,” (Schwartz, 2012, p.7; see also, Schwartz, 1992) has been linked to more favorable attitudes toward immigrants (Schwartz, 2007; Saroglou et al., 2009).

## Present Study

In the present study, we propose to test the idea that European identity (being a more inclusive high-order system-level or superordinate identity) should predict positive attitudes toward immigrants. Indeed, when a particular group membership is salient, the degree of ingroup identification predicts the adherence to that group’s norms and values (e.g., Tajfel and Turner, 1986). Thus, group identification represents a strong predictor of intergroup attitudes (Reysen and Katzarska-Miller, 2017). Given that EU supports values of solidarity, mutual respect, and inclusive policies and strategies, European identification should be contaminated by these values and predict high support for EU inclusive policies. Moreover, this superordinate identity (occurring in a higher level of abstraction than national identity) makes subgroup boundaries (e.g., nationality) less salient and some outgroups become a part of the broader ingroup (e.g., European citizens), leading to more favorable attitudes and behaviors toward migrants. Thus, identification with a superordinate identity supportive of an inclusive ideology predicts positive attitudes toward migrants (Visintin et al., 2018), prosocial behaviors, positive intergroup relations, cooperation, and social well-being (Reysen and Katzarska-Miller, 2013, 2017).

Based on the literature, we should expect national identity to predict contestation of immigrants’ inclusion. However, in these studies, national identity is often related to nationalism, an identity content that is consistent to right-wing ideology. People with a right-wing political affiliation usually have less favorable attitudes toward migrants than those with a left-wing affiliation (Sides and Citrin, 2007; Gorodzeisky and Semyonov, 2009; Green, 2009; Davidov and Meuleman, 2012). Right-wing individuals are expected to identify with this ideological content, but left-wing individuals are expected to embrace a national identity more open to diversity and equality. Thus, first of all, we propose that, depending on individuals’ political tendency, the link between national identity and anti-immigration attitudes might be opposite between left-wing and right-wing individuals. Left-wing individuals might value aspects of the national identity that are more related to pro-immigration attitudes (EU Inclusive Agenda). If so, left-wing individuals should positively associate national and European identity, whereas right-wing individuals are expected to associate them negatively.

Moreover, considering that national and European identities predict attitudes toward immigrants, we propose to inspect which arguments (found in political speeches and also in the literature) effectively persuade people to engage in contestation and support for exclusive policies, or in pro-immigrant behavior and support for inclusive policies. In brief, in the present study we intend to assess identity predictors of political discourses arguments and how it impacts contestation and prosocial behavioral intentions regarding immigrants’ entrance to Europe.

## Materials and Methods

### Participants and Procedure

Participants were contacted through Facebook (Facebook Ads, delivered randomly to Portuguese nationals above 18 years of age) and personal contacts to fill a survey about the “Increase of immigrants in Europe: What are the consequences.” This procedure began at November 11, 2019 and we kept the questionnaire active for a week. Participants who voluntarily decided to respond to the questionnaire are 176 Portuguese nationals (85 female and 91 male participants), aged 18–79 years (*M* = 40.06, *SD* = 14.02), living in Portugal (94%) or abroad (6%). The majority were employed (63%) and the remaining were students (11%), unemployed (9%), and retired (8%). Moreover, the majority (76%) of participants had higher education, whereas the remaining had secondary (21%) or basic (3%) education^1^.

Participation was completely voluntary and not monetarily compensated. After giving informed consent, participants provided their demographic information (e.g., age, sex, education, work status, political orientation).

### Instrument

Participants responded to seven sets of questions designed to assess identification with Portugal and the European Union, agreement with different types of political arguments (pro vs. anti-immigration), and agreement with inclusive and exclusive policies regarding immigrants, and reported their motivation to get involved in pro- (prosocial behavior) and anti-immigration (contestation) initiatives.

#### National Identity

The first set of questions measured participants’ identification with Portugal on a seven-point scale (1 = *Totally Disagree;* 7 = *Totally Agree*). To avoid associating an ideology to the national identity, we measured individuals’ identification based on Tajfel’s concept of social identification (Tajfel, 1978), inspired by the national identity scale in Pinto et al. (2016): (1) “I identify with Portuguese values and ideals”; (2) “I’m proud of being Portuguese”; (3) “I consider myself a Portuguese citizen”; (4) “Being Portuguese is important for my identity.” A Principal Component Factorial Analysis on these items extracted a single factor explaining 66.1% of the total variance. We averaged these items to a single National Identity score (Cronbach’s alpha = 0.83; *M* = 5.52; *SD* = 1.30).

#### European Identity

Identification with Europe was measured with a scale that was also inspired by the national identity scale in Pinto et al. (2016), but adapted to refer to European identity (1 = *Totally Disagree;* 7 = *Totally Agree*): (1) “I identify with European values and ideals”; (2) “I’m proud of being European”; (3) “I consider myself a European citizen”; (4) “Being European is important for my identity.” A Principal Component Factorial Analysis on these items extracted a single factor explaining 76.5% of the total variance. The items were averaged into a single European Identity score (Cronbach’s alpha = 0.89; *M* = 5.13; *SD* = 1.51).

#### Arguments

Next, participants indicated their agreement on five different types of arguments regarding the immigrants’ inflow (*1* = *Totally Disagree;* 7 = *Totally Agree*): (1) “The situation of immigrants should be seen as a humanitarian issue that is related with human rights” (humanitarian cause; *M* = 4.56; *SD* = 2.35); (2) “The situation of immigrants should be seen as being economically advantageous for the host countries” (economic benefit; *M* = 3.85; *SD* = 2.09); (3) “The situation of immigrants should be seen as an invasion” (invasion; *M* = 3.21; *SD* = 2.34); (4) “The situation of immigrants should be seen as competition for scarce jobs” (scarce jobs; *M* = 3.74; *SD* = 2.20); (5) “The situation of immigrants should be seen as a determinant for cultural deterioration” (cultural deterioration; *M* = 3.56; *SD* = 2.23). We analyzed each argument individually, since each item corresponds to a specific type or concept that is found in political speeches and in the literature as being associated to attitudes toward immigrants (e.g., realistic and symbolic threat: Stephan et al., 2005; universal values: Schwartz, 2007; invasion: Gattinara, 2017; de Wenden, 2019; loss of identity: Schmuck and Matthes, 2017).

#### Inclusive Policies

Participants’ agreement with European inclusive policies were measured with a seven-item scale (1 = *Totally Disagree;* 7 = *Totally Agree*): (1) “European countries should welcome and integrate immigrants arriving in Europe”; (2) “European countries should welcome all those who leave their own country and wish to live in Europe”; (3) “European countries should follow European guidelines on the inclusion of immigrants”; (4) “Europe earns plenty of money from immigrants’ contributions”; and three items adapted from the Immigration Policy Questionnaire (IPQ) (Tartakovsky and Walsh, 2016): (5) “Europe has the duty to provide free health care to all immigrants”; (6) “Immigrants should have free access to the same social services as European citizens.”; (7) “Immigrants should have free access to the same social security rights (e.g., elderly or disability pensions).” A Principal Component Factorial Analysis on these items extracted a single factor explaining 67% of the total variance. We averaged these items into an Inclusive Policies score (Cronbach’s alpha = 0.92; *M* = 4.47; *SD* = 1.45).

#### Exclusive Policies

Respondents reported their agreement with exclusive policies (1 = *Totally Disagree;* 7 = *Totally Agree*): (1) “European countries should have greater power to prevent immigrants from attempting to enter their territory”; (2) “European countries should be able to impose more restrictive border laws on the immigrants’ entry”; (3) “European countries should be free to prevent immigrants from entering, and not have to follow European guidelines”; (4) “Europe is unable to receive more immigrants”; (5) “Europe spends too much money in immigrant integration programs.” A Principal Component Factorial Analysis on these items extracted a single factor explaining 84% of the total variance. These items were averaged into a single Exclusive Policies score (Cronbach’s alpha = 0.95; *M* = 4.16; *SD* = 2.19).

#### Prosocial Behavior

Participants indicated their motivation to adopt prosocial behavior regarding immigrants. The first four items were adapted from Yitmen and Verkuyten (2018); (*1* = *I am not motivated at all; 7* = *I am very much motivated*): (1) “Helping an immigrant if s/he asks me to”; (2) “Participating in a march in favor of the immigrants’ integration in Europe”; (3) “Signing a petition in favor of immigrants’ integration in Europe”; (4) “Donating money to help immigrants”; (5) “Becoming a volunteer to help immigrants”; (6) “Participating in movements against nationalist movements”; (7) “Signing a petition to demand inclusive policies according to European indications”; (8) “Voting (on the 2024 European parliamentary elections) for political parties that support inclusive immigration policies.” A Principal Component Factorial Analysis on these items extracted a single factor explaining 70,8% of the total variance. We averaged these items into a Prosocial Behavior score (Cronbach’s alpha = 0.94; *M* = 3.64; *SD* = 1.91).

#### Contestation

Finally, participants indicated their motivation to participate in initiatives to contest immigrants’ integration in Europe (1 = *I am not motivated at all;* 7 = *I am very much motivated*): (1) “Protesting against the immigrants’ integration in Europe”; (2) “Signing a petition against the immigrants’ integration in Europe”; (3) “Protesting against European inclusive policies”; (4) “Voting (on the 2024 European parliamentary elections) for political parties that are against inclusive policies”; (5) “Voting (on the 2024 European parliamentary elections) for parties that defend ‘closed borders’ to immigrants.” A Principal Component Factorial Analysis on these items extracted a single factor explaining 85,3% of the total variance. We computed a Contestation score (Cronbach’s alpha = 0.97; *M* = 3.13; *SD* = 2.24) corresponding to the average of these items.

## Results

### The Association Between National Identity and European Identity

**FIGURE 1:**
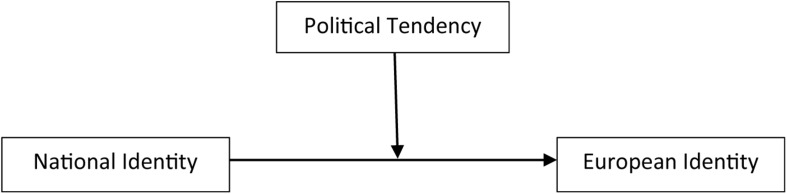
Moderation model, taking National Identity as predictor, European Identity as dependent variable and Political tendencies as moderator.

We propose that National Identity would show different patterns of association with European Identity, depending on individuals’ Political Tendency. In order to test this idea, we conducted a moderation analysis (using PROCESS 3.3 version, Model 1 with 10,000 bootstrap samples; Hayes, 2018), considering National Identity as the predictor, European Identity as the dependent variable, and Political Tendency as the moderator (see Figure 1).

This model explains 15% of the variability observed in European Identity (*F*3,172 = 9.75, *p* < 0.001). We observed that National Identification is, in general, associated with European Identity (*b* = 0.96, *p* < 0.001). We also observed that Political Tendency is associated with European Identity (*b* = 0.74, *p* = 0.002). Finally, and relevant to our hypothesis, we also observed a significant National Identity × Political Tendency (*b* = −0.16, *p* < 0.001). The analysis splits Political Tendency in three groups based on Mean ± 1 SD and examines the association between both identities within each political tendency. We denominated those groups as left-wing (*M* = 2.04; presenting scores below one standard deviation below the mean), center (*M* = 3.84; corresponding to scores between M−SD and M+SD), and right-wing (*M* = 5.63; presenting scores above one standard deviation above the mean). As predicted, results show different patterns of association between National Identity and European Identity depending on individuals’ political tendency. We observed significant and positive associations between both identities in the left-wing and center group (*b* = 0.63, *SE* = 0.12, *t* = 5.24, *p* < 0.001 and *b* = 0.34, *SE* = 0.09, *t* = 4.01, *p* < 0.001, respectively), and no significant association in the right-wing group (*b* = 0.05, *SE* = 0.11, *p* = 0.640). These results highlight that, for the left and center Political Tendency, the more individuals identify with Portugal, the more they identify with Europe. Both identities seem to be independent from each other for right-wing participants.

### Identities as Predictors of Positive and Negative Arguments for Immigrants’ Entrance

**FIGURE 2:**
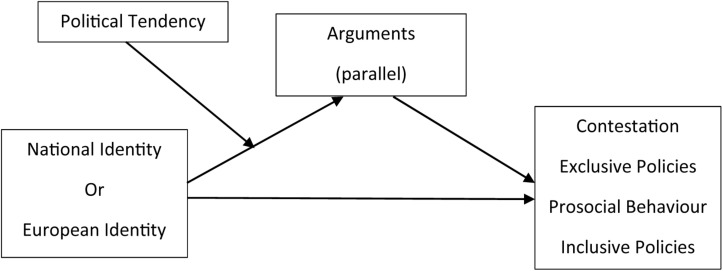
Moderated mediation models (PROCESS 3.3 version, Model 7), taking National Identity or European Identity as predictor, Contestation, Exclusive Policies, Prosocial Behavior and Inclusive Policies, Contestation or Prosocial Behavior as dependent variable, Arguments (Humanitarian; Economic Benefit; Scarce Jobs; Cultural Deterioration; Invasion) as parallel mediators (included in all models) and Political Tendency as the moderator (included in all models).

We hypothesize that National and European Identities might rely on different arguments to predict negative (Contestation and Exclusive Policies) or positive (Prosocial Behavior and Inclusive Policies) attitudes toward immigrants. In order to test this idea, we conducted four moderated mediation models (using PROCESS 3.3 version, Model 7, with 10,000 bootstrap samples; Hayes, 2018) by each type of Identity (National or European), for a total of eight models. All models considered (National or European) Identity as predictor, the five arguments (humanitarian; economic benefit; scarce jobs; cultural deterioration; invasion) as parallel mediators, and Political Tendency as the moderator. One model considered Contestation, another model considered Exclusive Policies, another one considered Prosocial Behavior, and the final one considered Inclusive Policies as the dependent variable; see Figure 2.

Before reporting the direct and indirect (through positive and negative arguments) predictive role of identities on Contestation, Exclusive Policies, Prosocial Behavior, and Inclusive Policies, we will describe the effect of National and European Identity, of Political Tendency, and of National and European Identity × Political Tendency to predict support for each argument; that is, the first stage of the moderated mediation model.

#### Humanitarian Argument

National Identity (*b* = 0.79, *p* < 0.001) significantly predicts perception of immigrants’ entrance as a humanitarian concern. European Identity did not predict this argument (*b* = 0.10, *p* = 0.464). We found a significant National Identity × Political Tendency (*b* = −0.17, *p* = 0.005) but not European Identity × Political Tendency (*b* = 0.07, *p* = 0.130) interaction on Humanitarian argument. Interestingly, results show that only left-wing participants (*b* = 0.43, *p* = 0.014) seem to relate national identity with the Humanitarian argument; the more they identify with Portugal, the more they perceive immigrants’ entrance as a humanitarian concern.

#### Economic Benefit Argument

National Identity (*b* = 0.40, *p* = 0.121) and European Identity (*b* = −0.16, *p* = 0.410) did not directly predict perception of immigrants’ entrance as being economically benefiting. We found significant National Identity × Political Tendency (*b* = −0.11, *p* = 0.050) and European Identity × Political Tendency (*b* = 0.10, *p* = 0.020) interactions on the Economic Benefit argument. Results show that European Identity is positively related with Economic Benefit argument among center (*b* = 0.23, *p* = 0.015) and right-wing (*b* = 0.41, *p* = 0.001) participants. Regarding National Identity, results show a tendency to opposite patterns of association between left and right-wing participants, although not significant.

#### Scarce Jobs Argument

National Identity (*b* = −0.16, *p* = 0.594) and European Identity (*b* = 0.24, *p* = 0.298) did not directly predict perception of immigrants’ entrance as being a threat to national citizens’ jobs. We found a marginally significant European Identity × Political Tendency (*b* = −0.08, *p* = 0.091) on the Scarce Jobs argument but not a National Identity × Political Tendency interaction (*b* = 0.03, *p* = 0.692). Results show a marginally significant effect only among right-wing participants (*b* = −0.24, *p* = 0.074): the more they identify with Europe, the less they perceive immigrants as a threat to nationals’ jobs.

#### Cultural Deterioration Argument

National Identity (*b* = −0.44, *p* = 0.060) marginally predicts this argument, contrary to the European Identity (*b* = 0.14, *p* = 0.491). We found significant National Identity × Political Tendency (*b* = 0.16, *p* = 0.006) and marginally significant European Identity × Political Tendency (*b* = −0.08, *p* = 0.061) interactions on the Cultural Deterioration argument. Results show that European Identity is negatively related with the Cultural Deterioration argument among center (*b* = −0.18, *p* = 0.059) and right-wing (*b* = −0.34, *p* = 0.006) participants, such that the more these groups identify with Europe, the less they perceive immigrants as deteriorating the host culture. Regarding National Identity, results show the opposite pattern of association within right-wing participants (*b* = 0.45, *p* = 0.002; for the remaining groups, results are non-significant): the higher the national identification, the higher the perception that immigrants deteriorate the host culture.

#### Invasion Argument

National Identity (*b* = 0.20, *p* = 0.034), but not European Identity (*b* = 0.07, *p* = 0.754), directly predicts perception of immigrants’ entrance as an invasion. We found a significant National Identity × Political Tendency (*b* = 0.20, *p* < 0.001) interaction on the Invasion argument (European Identity × Political Tendency: *b* = −0.07, *p* = 0.753). The more right-wing (*b* = 0.54, *p* = 0.001; for the remaining groups results are non-significant) participants identify with Portugal, the more they perceive immigrants as invaders.

Taken together, results showed that among left-wing participants, National Identity positively predicts Humanitarian argument (European Identity was not a significant predictor of any of the proposed arguments in this political tendency, although National identity is strongly related to European identity among left-wing participants, as shown in the previous analysis). For those participants positioned in the center, National Identity did not emerge as a predictor of any of the arguments. However, European Identity positively predicts the Economic Benefit argument, and negatively the Cultural Deterioration argument. Finally, among the right-wing participants, National Identity positively predicts Cultural Deterioration and Invasion arguments. European Identity negatively predicts Cultural Deterioration and Scarce Jobs arguments, and positively the Economic Benefit argument.

Thus, those who relate national identification to the humanitarian argument are positioned on the left of the political spectrum. These participants aligned National Identity with EU inclusive values of solidarity and mutual respect. Inversely, the association found among right-wing participants, national identification and cultural deterioration and invasion arguments seem more aligned with nationalist beliefs and protective attitudes (i.e., beliefs in the superiority of own nation associated with hostility directed to immigrants). Nevertheless, and still among the right-wing participants, the more they identify with Europe (i.e., more inclusive group with an inclusive ideology), the less sensitive they are to arguments of scarce jobs and cultural deterioration, and the more they believe in the economic benefits of immigrants. In other words, identification with Europe seems to counteract the effect of nationalist and protective attitudes on right-wing participants’ sensitivity to arguments of scarce jobs and cultural deterioration.

### Moderated Mediation Analyses

#### Contestation

The analysis on Contestation considering National Identity as the predictor (and arguments as mediators) shows that only Humanitarian (Moderated mediation index = 0.04, *SE* = 0.02, *95% CI* ]0.00, 0.09]) and Invasion (Moderated mediation index = 0.07, *SE* = 0.03, *95% CI* [0.02, 0.13]) arguments emerged as significant mediators of the association between National Identity and Contestation. The remaining arguments showed results with *95% CI* including 0 (that is, non-significant). A further inspection shows that left-wing participants (*b* = −0.10, *95% CI* [−0.23, −0.01]) are sensitive to the Humanitarian argument, such that the higher their national identification, the more they perceive immigrants’ entrance as a humanitarian concern and thus, the less they support contestation. Concomitantly, right-wing (*b* = 0.20, *95% CI* [0.07, 0.35]) participants are more sensitive to the Invasion argument: the more they identify with Portugal, the more they believe immigrants are invaders, and the more they support contestation.

If we take European Identity as the predictor, the Economic Benefit argument is the only one that emerged as a significant mediator (Moderated mediation index = −0.03, *SE* = 0.01, *95% CI* [−0.06, −0.00[). Nevertheless, although Humanitarian (Moderated mediation index = −0.01, *SE* = 0.01, *95% CI* [−0.05, 0.01]) and Invasion (Moderated mediation index = −0.03, *SE* = 0.02, *95% CI* [−0.07, 0.01]) did not emerge as significant mediators (*95% CI*, includes 0), some partial mediations are significant. The more center and right-wing participants identify with Europe, the more they believe immigrants can be economically benefiting (center: *b* = −0.06, *95% CI* [−0.13, −0.01]; right-wing: *b* = −0.11, *95% CI* [−0.20, −0.04]) and a humanitarian concern (center: *b* = −0.08, *95% CI* [−0.16, −0.02]; right-wing: *b* = −0.10, *95% CI* [−0.21, −0.03]); the less they believe they are invading Europe (center: *b* = −0.08, *95% CI* [−0.18, −0.00[; right-wing: *b* = −0.10, *95% CI* [−0.25, −0.03]), and consequently, the less they support contestation.

#### Exclusive Policies

The analysis on agreement with Exclusive Policies considering National Identity as the predictor shows that only Cultural Deterioration (Moderated mediation index = 0.03, *SE* = 0.02, *95% CI* [0.00, 0.06] – marginally significant) and Invasion (Moderated mediation index = 0.07, *SE* = 0.03, *95% CI* [0.03, 0.13]) arguments emerged as mediators of the association between National Identity and Exclusive Policies (*95% CI* for remaining arguments includes 0). A further inspection shows that right-wing participants are sensitive to these arguments, such that the more they identify with Portugal, the more they believe immigrants cause cultural deterioration (*b* = 0.07, *95% CI* ]0.00, 0.16]) and are invaders (*b* = 0.20, *95% CI* [0.08, 0.33]), and the more they support exclusive policies.

Regarding European Identity (predictor), besides the above arguments being marginally significant (Cultural Deterioration and Invasion: Moderated mediation index = −0.01, *SE* = 0.01, *95% CI* [−0.03, 0.00] and Moderated mediation index = −0.03, *SE* = 0.02, *95% CI* [−0.07, 0.00], respectively), the Economic Benefit argument emerged as a significant mediator (Moderated mediation index = −0.04, *SE* = 0.02, *95% CI* [−0.08, −0.01]; *95% CI* for the remaining arguments includes 0). Center and especially right-wing participants show that the more they identify with Europe, the more they believe immigrants can be economically benefiting (center: *b* = −0.08, *95% CI* [−0.17, −0.02]; right-wing: *b* = −0.15, *95% CI* [−0.26, −0.07]) and the less they believe they cause cultural deterioration (right-wing: *b* = −0.05, *95% CI* [−0.12, −0.01]) and are invading Europe (right-wing: *b* = −0.12, *95% CI* [−0.24, −0.03]; these last two arguments only for right-wing participants); thus, the less they support exclusive policies.

#### Prosocial Behavior

The analysis on Prosocial behavior considering National Identity as the predictor shows that only Invasion (Moderated mediation index = −0.03, *SE* = 0.02, *95% CI* [−0.05, −0.00[) emerged as significant mediator of the association between National Identity and Prosocial behavior (remaining arguments present *95% CI* including 0). For right-wing participants (*b* = −0.09, *95% CI* [−0.18, −0.01]), the higher their national identification, the more they perceive immigrants as invaders and the less they support prosocial behavior.

Regarding European identity as the predictor, the Economic Benefit argument (Moderated mediation index = 0.05, *SE* = 0.02, *95% CI* [0.01, 0.09]) and in a marginally significant way the Invasion argument (Moderated mediation index = 0.01, *SE* = 0.01, *95% CI* [0.00, 0.04]) emerged as mediators (for the remaining arguments, *95% CI* include 0). Center and right-wing participants show that the more they identify with Europe, the more they believe immigrants can be economically benefiting (center: *b* = 0.10, *95% CI* [0.02, 0.20]; right-wing: *b* = 0.19, *95% CI* [0.08, 0.33]) and the less they believe they are invading Europe (only for right-wing: *b* = 0.06, *95% CI* ]0.00, 0.13]), thus the more they feel motivated to get involved in pro-immigration behavior.

#### Inclusive Policies

The analysis on support for inclusive policies considering National Identity as the predictor shows that Cultural Deterioration (Moderated mediation index = −0.02, *SE* = 0.01, *95% CI* [−0.05, −0.00[) and Invasion (Moderated mediation index = −0.02, *SE* = 0.01, *95% CI* [−0.05, −0.00[) emerged as significant mediators of the association between National Identity and Inclusive Policies. Right-wing participants, again, are those that are most sensitive to these arguments: the stronger their national identification, the more they perceive immigrants as causing cultural deterioration (*b* = −0.05, *95% CI* [−0.13, −0.00[) and as invaders (*b* = −0.06, *95% CI* [−0.14, −0.00[), and the less they support inclusive policies (remaining arguments present *95% CI* including 0).

Considering European identity, the Economic Benefit argument (Moderated mediation index = 0.02, *SE* = 0.01, *95% CI* ]0.00, 0.04]) and, in a marginally significant way, the Cultural Deterioration (Moderated mediation index = 0.01, *SE* = 0.01, *95% CI* [−0.00, 0.03]) and Invasion (Moderated mediation index = 0.01, *SE* = 0.01, *95% CI* [−0.00, 0.03]) arguments emerged as mediators in the analysis (remaining arguments present 95% CI including 0). Center and right-wing participants show that the more they identify with Europe, the more they believe immigrants can be economically benefiting (center: *b* = 0.04, *95% CI* [0.01, 0.09]; right-wing: *b* = 0.08, *95% CI* [0.03, 0.14]), and the more they support inclusive policies. Right-wing participants show the opposite pattern for Cultural Deterioration (*b* = 0.04, *95% CI* [0.01, 0.10]) and Invasion (*b* = 0.04, *95% CI* ]0.00, 0.09]).

## Discussion

National Identity, European Identity and Political Tendency emerged as crucial predictors of anti- and pro-immigrant attitudes.

### Political Tendency and Attitudes Toward Immigrants

First of all, our results show that right-wing political tendency is associated to more exclusive policies and contestation and less inclusive policies and prosocial behavior, whereas left-wing political tendency is more associated to the opposite attitudes.

### National and European Identity

Left-wing participants positively associate National with European Identity, reflecting that, among these participants, both identities seem to assume similar ideological contents. On the contrary, right-wing participants do not relate National to European Identity. Moreover, for this group of participants, National and European Identities show opposite patterns of prediction regarding some of the arguments and attitudes regarding immigrants: National Identity is more supportive of contestation and exclusive policies, whereas European Identity, on the contrary, is more predictive of pro-immigration attitudes, both regarding prosocial behavior and support for inclusive policies.

Interestingly, the above effects are mainly found among right-wing participants, suggesting that these participants may be those most susceptible to be influenced by both social identities.

### Arguments

We tested the mediating effect of two positive (humanitarian concern and economic benefit) and three negative (scarce jobs, cultural deterioration, and invasion) arguments on the relationship between national identity vs. European identity and supporting immigrants’ inclusion vs. immigrants’ exclusion.

The humanitarian argument emerged as significant for decreasing contestation. For left-wing participants, National Identity predicts low levels of COntestation through Humanitarian arguments. The same pattern occurs for right-wing participants, but regarding European Identity instead of National Identity.

Economic benefit emerged as a relevant mediator between European Identity (but not National Identity) and (positive and negative) attitudes toward immigrants, but only among right-wing participants.

The negative arguments that are more relevant to predicting attitudes toward immigrants are Invasion and Cultural Deterioration, though only among right-wing participants. Among these participants, National Identity predicts a conservative and discriminatory pattern of association, showing that the more right-wing participants identify with Portugal, the more they adhere to arguments of invasion and cultural deterioration of the host society and, consequently, the more they agree with negative, and less with positive, attitudes toward immigrants. On the contrary, European Identity predicts a liberal pattern of association: the more right-wing participants identify with Europe, the less they believe immigrants are invaders and culturally harmful, and the more they engage in pro-immigrant attitudes (and less in anti-immigrant attitudes).

## Conclusion

Literature has shown that national identity, associated with nationalist sentiments, predicts stronger negative attitudes toward immigrants and greater opposition to immigration. Our results suggest that national identity predicts negative attitudes toward immigrants, but only among right-wing orientation individuals. On the contrary, among left-wing participants, national identity predicted less contestation to immigrants’ integration. In our study, right-wing participants were more sensitive to arguments focused on immigrants’ invasion and cultural deterioration impact, whereas left-wing participants were more sensitive to humanitarian concerns. These arguments’ differentiation emerged as significant mediators of the association between national identity and attitudes toward immigrants: whereas the more right-wing participants identify with Portugal, the more they perceive immigrants as invaders that may deteriorate their culture; for the left-wing participants, the more they identify with Portugal, the more they think immigrants’ entrance is a humanitarian concern. These results suggest that national identity is not a consensual homogeneous identity and it seems to leave room for diversity for left-wing participants, whereas it seems to enact protective attitudes among right-wing participants (consistent with Frederic and Falomir-Pichastor, 2018).

In our study, we also tested the predictive effect of participants’ identification with the European Union on participants attitudes toward immigrants. Evidence has shown that by encompassing values of solidarity, mutual respect, and humanitarian concerns, identification with European Union predicts support for inclusive policies and the emergence of positive attitudes toward immigrants. Nevertheless, this process was found only among right-wing oriented individuals. Interestingly, this process seems to suggest that identification with the European Union weakens right-wing individuals’ adherence to arguments favoring the discrimination of immigrants (cultural deterioration and invasion), while increasing the acceptance of immigrants’ economic contribution to the host society, leading to a decrease of the agreement with exclusive policies and contestation of immigrants in Europe. These results suggest that right-wing participants value the positive contribution that immigrants bring to the European Union economy, and that this argument might be a relevant justification for immigrants’ acceptance by this group. This seem to be a potential response to the economic security concerns that civil society (and namely those more skeptic about EU) has been debating regarding Europe (Wodak and Boukala, 2015). Indeed, there is evidence that recognizing the economic indispensability that immigrants may represent leads to more positive attitudes toward them (Guerra et al., 2015).

Interestingly (and curiously), left-wing participants did not show any connection between identification with European Union and positive attitudes toward immigrants. Although national identity is correlated to European identity for this social group, the fact is that national identity seems more relevant to influencing individuals’ attitudes regarding immigrants.

In brief, the present results may contribute two relevant pathways for the promotion of inclusive societies and contribution for a positive social integration of immigrants, inclusive social cohesion and, thus, more societal well-being, anchored in pro-diversity identities, and reinforced prosocial behavior anchored in stronger and more resilient societies (Nerone, 2019; Randall et al., 2019).

First, because European Union has a clear ideological content that is in line with pro-inclusive societies, it is expected that identification with European Union leads to more positive attitudes toward immigrants. Thus, it would be important to promote national identities’ ideology closer to the European ideological content. One possible direction is the one shown by the “Ingroup Projection Model” (Mummendey and Wenzel, 1999). According to this model, it is possible to reduce conflicting attitudes toward the outgroup (in this case, immigrants) through the interaction of two mechanisms: (1) Dual identification – when both (national and European) identities are frequently salient and thus equally cognitively accessible to define the self, both become correlated in individuals’ mind after a while; (2) Complexity – by being ideologically pro-diversity, the European Union identity value several representative ingroup members (multimodal prototype), thus decreasing ethnocentricity. Left-wing participants already correlate both identities, but among them, national identity seems to be more relevant to determine attitudes toward immigrants. Once our results showed that National and European Identities determined more right-wing participants’ (than left-wing participants’) attitudes toward immigrants by improving dual identification process among right-wing individuals, both identities should become more interrelated and should embrace pro-diversity ideology. As a result, this process should contribute to counteract right-wing individuals’ tendency to be less favorable toward migrants when their national identity is salient.

Second, our results indicate that the arguments that seem more effective in predicting positive attitudes toward immigrants among right-wing individuals are immigrants’ economic contribution to the host society. On the other hand, the arguments that most predict negative attitudes toward immigrants are their supposed impact on cultural deterioration and invasion (which reflects ethnocentrism). These results indicate that the approach of facts related to these arguments in political discourses might influence public opinion. In order to promote pro-inclusion attitudes and decrease blatant ethnocentrism, political discourses should present facts that show the economic benefit brought by immigrants and, simultaneously, facts that decrease the perceived threat of cultural deterioration and invasion on the part of immigrants.

### Limitations and Future Research

Although the aim for this study did not require the collection of a representative sample, the fact is that our sample is biased regarding participants’ education level. Because this variable is found in the literature as having impact on individual political attitudes, it would be of extreme relevance to test these processes in more diverse samples.

This study focused on the impact of national and European identities on attitudes regarding immigrants. We tested how both identities were associated with each other and with attitudes toward immigrants. Results were consistent with the assumption that European Identity content should be oriented by the inclusive European Agenda values. Nevertheless, we did not inspect the actual content of each identity by political tendency, which would be relevant. For instance, literature highlights that confidence in EU institutions is determinant to the European identity definition (Verhaegen et al., 2017). If so, a higher skepticism regarding EU might decrease the chances of being able to strengthen a European identity relying on EU inclusive values and foster anti-immigration attitudes (and even nationalisms).

Finally, as a future direction, immigrants’ nationality might play a big role in these processes. Traditionally, immigrant communities in Portugal come from ex-colonies, but Portugal has more recently being receiving immigrants from other nationalities. Ethnocentrism regarding immigrants is also fueled by stereotypes associated to immigrants’ groups of origin (Lee and Fiske, 2006), which potentiates the interest in investigating the role of immigrants’ nationality as a moderator of these processes (Kentmen-Cin and Erisen, 2017).

## Data Availability Statement

The raw data supporting the conclusions of this article will be made available by the authors, without undue reservation.

## Ethics Statement

Ethical review and approval was not required for the study on human participants in accordance with the local legislation and institutional requirements. The patients/participants provided their written informed consent to participate in this study.

## Author Contributions

All authors listed have made a substantial, direct and intellectual contribution to the work, and approved it for publication.

## Conflict of Interest

The authors declare that the research was conducted in the absence of any commercial or financial relationships that could be construed as a potential conflict of interest.

## Footnotes

1 Sociodemographic variables also contribute to understand individuals’ attitudes toward immigrants, such as gender, age, education and political affiliation (e.g., Coenders and Scheepers, 2003). Nevertheless, in this study we will only focus on the role of individuals’ political tendencies in predicting these attitudes.
